# Primary eye health care: the foundation for universal access to eye health

**Published:** 2022-03-01

**Authors:** Elmien Wolvaardt, Kriti Shukla

**Affiliations:** 1 Editor: *Community Eye Health Journal*, International Centre for Eye Health, London School of Hygiene & Tropical Medicine, London, UK.; 2 Editor: *Community Eye Health Journal*, South Asia edition Research Associate – Centre for Health Outcomes Research and Economics, Indian Institute of Public Health, Hyderabad, India.

**The majority of eye conditions should ideally be dealt with at primary health care level – in the communities where people live and work. However, eye care is often missing or not integrated within primary health care – a challenge this issue wants to address.**

**Figure:**
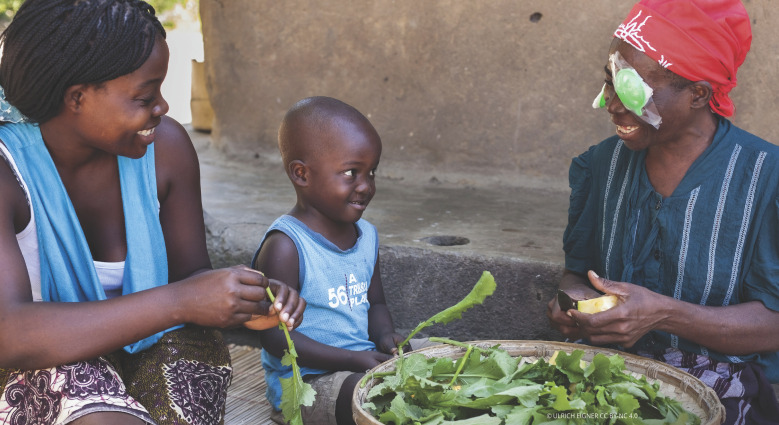
Primary eye health care workers can detect, counsel, and refer patients with cataract to the nearest eye unit. **MOZAMBIQUE**

In low-resource settings, almost 80% of the causes of blindness and visual impairment are avoidable,^1^ as they can be prevented, or treated to restore vision. Alongside treatment and referral, it is therefore vital that eye care at primary level includes health promotion – working with communities and policy makers to create environments that support better eye health, teaching people how to take care of their eyes, and telling them what to do (and where to go) when they have an eye problem that cannot be treated by primary health care workers.

When eye care is not available at primary health care level, for whatever reason, the secondary/district level or tertiary/teaching hospital is the first place where people with eye problems seek care – often after waiting until their eye condition has worsened or after seeking unconventional treatment (such as from traditional healers), with negative results.

By creating policies that integrate eye health care within primary health care, and supporting local primary health teams to provide high quality eye care for simple eye conditions (referring only those patients who need further care), we can help to reduce the load on outpatient clinics in secondary and tertiary level facilities – thereby making the eye health system more efficient. Primary eye health care also ensures that patients can get the right care quickly, without facing the cost or other barriers associated with travelling long distances, such as loss of daily wages, lack of transportation, or having to find someone to take over their caring responsibilities. It is worth noting that, in the absence of care at primary level, the most underserved groups – women, people in poverty, and those with disabilities – are disproportionally disadvantaged.

Effective primary eye health care provision requires an integrated approach, with supportive policies and adequate funding, such as appropriate and sustainable training and support for the health workers who deliver eye care at this level, and integrated health information systems to ensure that progress is measured and maintained and so that patients can be effectively referred and followed up.

This issue shares best practices and approaches for integrating eye care within primary health care, and we have included guidance about what eye care professionals can do. But it is just as important that decision makers and policy makers at all levels are aware of the need for primary eye health care and what they can do to support it. We therefore encourage you to share this issue with policy makers and decision makers at local, regional, and national level: their active involvement is needed in order to ensure universal access to eye health care for all.
